# Dopaminergic Modulation of Goal-Directed Behavior in a Rodent Model of Attention-Deficit/Hyperactivity Disorder

**DOI:** 10.3389/fnint.2018.00045

**Published:** 2018-10-05

**Authors:** Joman Y. Natsheh, Michael W. Shiflett

**Affiliations:** ^1^Center for Molecular and Behavioral Neuroscience, Rutgers University, Newark, NJ, United States; ^2^Kessler Foundation, East Hanover, NJ, United States; ^3^Palestinian Neuroscience Initiative, Al-Quds University, East Jerusalem, Palestine; ^4^Children’s Specialized Hospital Research Center, New Brunswick, NJ, United States; ^5^Department of Psychology, Rutgers University, Newark, NJ, United States

**Keywords:** attention deficit hyperactivity disorder, spontaneous hypertensive rats, Wistar-Kyoto rats, goal-directed behavior, habitual behavior, action control, dopamine

## Abstract

Aside from its clinical symptoms of inattention, impulsivity and hyperactivity, patients with Attention/Deficit-Hyperactivity Disorder (ADHD) display reward and motivational impairments. These impairments may reflect a deficit in action control, that is, an inability to flexibly adapt behavior to changing consequences. We previously showed that spontaneously hypertensive rats (SHR), an inbred rodent model of ADHD, show impairments in goal-directed action control, and instead are predominated by habits. In this study, we examined the effects of specific dopamine receptor sub-type (D1 and D2) agonists and antagonists on goal-directed behavior in SHR and the normotensive inbred control strain Wistar-Kyoto (WKY) rats. Rats acquired an instrumental response for different-flavored food rewards. A selective-satiety outcome devaluation procedure followed by a choice test in extinction revealed outcome-insensitive habitual behavior in SHR rats. Outcome-sensitive goal-directed behavior was restored in SHR rats following injection prior to the choice test of the dopamine D2 receptor agonist Quinpirole or dopamine D1 receptor antagonist SCH23390, whereas WKY rats showed habitual responding following exposure to these drugs. This novel finding indicates that the core behavioral deficit in ADHD might not be a consequence of dopamine hypofunction, but rather is due to a misbalance between activation of dopamine D1 and D2 receptor pathways that govern action control.

## Introduction

Attention-Deficit/Hyperactivity Disorder (ADHD) is one of the most prevalent psychiatric disorders, characterized by developmentally inappropriate symptoms of inattention, hyperactivity and impulsivity (Castellanos and Tannock, 2002; Barkley, 2005). Along with these symptoms, individuals with ADHD show motivational impairments (Carlson and Tamm, 2000; Konrad et al., 2000; Slusarek et al., 2001; Castellanos and Tannock, 2002; McInerney and Kerns, 2003; Tripp and Wickens, 2008; Luman et al., 2010). For example, children with ADHD show reduced sensitivity to positive reinforcement compared to healthy children, they fail to adapt appropriately to changing rates of reinforcement, and they require larger incentives to adjust their actions (Tripp and Wickens, 2008; Volkow et al., 2011). Despite this evidence, the nature of reward processing deficits in ADHD has not been fully characterized. For example, reduced task motivation in ADHD could reflect blunted reward sensitivity, a failure to encode action-reward contingencies, or impaired action control. The latter process, action control, refers to the way in which voluntary actions are selected and executed based on prior reinforcement learning. Behavioral, computational and neural evidence identifies two parallel, interactive systems that underlie action control: a goal-directed system for deliberative actions, and a habitual system for reflexive actions (Tolman and Gleitman, 1949; Dickinson, 1985; Balleine and Dickinson, 1998; Dayan and Balleine, 2002; Balleine and O’Doherty, 2010). Importantly, goal-directed and habitual systems place different demands on attentional resources (Hitchcott et al., 2007; Le Pelley et al., 2013; Savalia et al., 2016; Luque et al., 2017), and as a consequence individuals with ADHD may be biased toward habitual action control.

Brain regions responsible for action control (e.g., cortico-basal ganglia circuitry), as well as dopamine signaling within these regions, show abnormalities in ADHD (Castellanos et al., 2006; Volkow et al., 2011; Furukawa et al., 2014; Hauser et al., 2014; von Rhein et al., 2015). Dopamine plays a critical role in action selection and initiation; therefore, a deficit in action control in ADHD may arise, in part, from misbalanced dopamine signaling within the basal ganglia (Yin et al., 2008; Tripp and Wickens, 2009). In our previous study (Natsheh and Shiflett, 2015), we found that spontaneously hypertensive rats (SHR), a rat model of ADHD, show a deficiency in goal-directed behavior that is restored by methylphenidate, which increases dopamine signaling. Studies in healthy rats have shown that exposure to amphetamine (Nelson and Killcross, 2006; Nordquist et al., 2007), alcohol (Corbit et al., 2012), stress (Dias-Ferreira et al., 2009) or binge-like consumption of a palatable food (Furlong et al., 2014) can accelerate habitual control. Interestingly, systemic or intra-striatal administration of D1 receptor antagonists (SCH23390) restored goal-directed behavior in these animals, whereas D2 receptor antagonists (Eticlopride) enhanced habitual behavior (Nelson and Killcross, 2013; Furlong et al., 2014). Taken together, these findings suggest normal patterns of goal-directed behavior rely on optimal activity levels of D1 and D2 dopamine receptors (D1R, D2R). Over-activation of D1R or under-activation of D2R could disrupt goal-directed behavior and/or promote habitual behavior.

**Figure 1:**
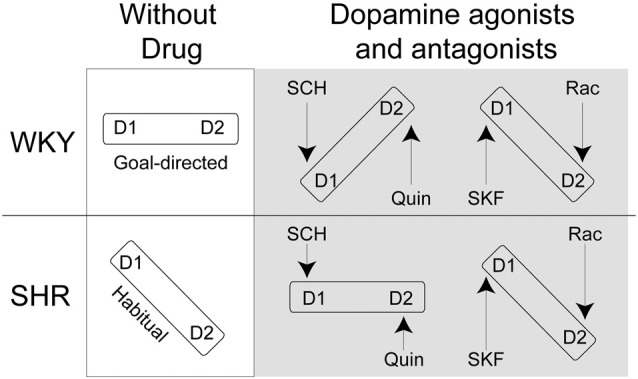
Theoretical framework for D1R/D2R balance in Spontaneously Hypertensive Rats (SHR) and Wistar-Kyoto (WKY) rats following normal saline, SCH23390 (SCH), Quinpirole (Quin), SKF38393 (SKF) and Raclopride (Rac) injections. If D1R and D2R are in balance we expect that rats will show goal-directed behavior. If D1R and D2R are not in balance, we expect that rats will show habitual response.

Here, we hypothesize that impaired goal-directed behavior in SHR rats results from a misbalance in D1R and D2R activation. We assessed the effects of the D1R agonist SKF38393, and antagonist SCH23390, and D2R agonist Quinpirole and antagonist Raclopride on goal-directed behavior in SHR rats and their control strain, Wistar Kyoto (WKY) rats. To assess action control, we used instrumental procedures in which rats made responses to gain access to rewarding food pellets. We then used a selective-satiety procedure to examine whether rats used outcome value to guide behavior, i.e., whether their behavior was goal-directed. We predict that the D1R antagonist SCH23390 and the D2R agonist Quinpirole will restore goal-directed behavior in SHR rats and impair this behavior in control rats. We expect the opposite set of agonist/antagonist pairings (SKF38393/Raclopride) will further impair performance in SHR rats as well as in control rats (Figure 1).

## Materials and Methods

### Subjects and Apparatus

Seventy-two male adult (P49–P80) rats were used in this study; 36 of which were SHR (ADHD rat model) from Charles River Laboratories (Wilmington, MA, USA), and 36 were WKY rats, the normotensive control strain, from Envigo (Indianapolis, IN, USA). The choice of strains from different vendors is based on previous studies showing that the WKY strain from Envigo (formerly, Harlan) is most similar genetically to Charles River SHR rats (Sagvolden and Johansen, 2012). The choice of age is based on the notion that SHR rats can start to develop symptoms of hypertension between the ages of 4–10 weeks which can result in neurological and behavioral deficits (Marcil et al., 1997; Christiansen et al., 2002; Ueno et al., 2002). Therefore, the age that has been widely used across SHR studies and that offers the fewest complications with hypertension is younger adult rats to serve as a model for ADHD (Sagvolden et al., 1992, 1993, 1998; Sagvolden, 2000). Rats weighed approximately 110–175 g at the time of testing.

Rats were housed in pairs in 47.6 × 20.3 × 26 cm (w × h × d) polycarbonate containers with Alpha Chip bedding material (Northeastern Products Corp., Warrensburg, NY, USA) and had free access to water. One week after arrival, all rats were placed on a restricted food diet of approximately 15 g of standard rat pellets (Purina, St. Louis, MO, USA) per day. Rats were fed after their daily behavioral training session. Food restriction continued for the duration of the experiment. All procedures were approved and carried out in accordance with the recommendations of the Rutgers University Institutional Animal Care and Use Committee.

Behavioral training and testing took place in 12 identical rat operant conditioning chambers (Med Associates, St. Albans, VT, USA). Each operant conditioning chamber measured 30.5 × 24.1 × 21 cm (w × h × d) and was constructed of stainless steel and clear plastic walls and a stainless-steel grid floor. A food cup with infrared detectors was centered on one wall of the operant conditioning chamber. Retractable levers were situated to the left and right of the food cup. Responses on these levers delivered one food pellet from a pellet dispenser mounted outside the operant conditioning chamber. Two types of pellets were used in the experimental procedures: 45-mg grain-based pellets and chocolate-flavored purified pellets (Bio-serv, Frenchtown, NJ, USA). Each operant conditioning chamber was housed in a sound attenuating shell and equipped with a ventilation fan that was activated during behavioral procedures. Control over the operant conditioning chambers was enabled by a personal computer operating through an interface. Operant conditioning chamber operation and data collection were carried out with Med Associates proprietary software (Med-PC).

### Behavioral Procedures

#### General Procedures

**Figure 2:**
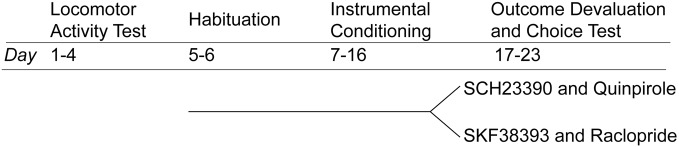
A timeline for behavioral experiments.

A description/timeline of behavioral procedures is depicted in Figure 2. Behavioral procedures commenced after 1 week of food restriction. After instrumental training, rats were divided into two groups. One group received injections of saline, SCH23390 and Quinpirole. The second group received injections of saline, SKF38393 and Raclopride.

#### Instrumental Conditioning

Rats underwent two training sessions per day. For each training session, one lever was inserted into the chamber and responses the rats made on the lever delivered a single 45-mg food pellet (Bio-serv, Flemington, NJ, USA). Responses on one lever delivered chocolate-flavored pellets, and the opposite lever delivered grain-based pellets. Rats were trained daily on each lever in separate sessions with a 30-min interval between sessions. The session terminated when rats earned 20 pellets or 25 min had elapsed. Training lasted for 10 days (see Figure 2); on days 1–3, each response on the lever resulted in pellet delivery (continuous reinforcement). On days 4–5, pellets were delivered according to a variable-ratio (VR) 5 schedule, which required, on average, five responses to earn a pellet reward. On days 6–8, pellets were delivered according to a VR-15 schedule. On days 9–10, pellets were delivered according to a VR-20 schedule.

#### Outcome Devaluation Test and Drug Injection

Rats were placed in individual cages identical to their home cage and provided with 25 g of one of the instrumental outcomes (either grain or chocolate-flavored pellets). After 40 min, rats were given an intraperitoneal injection of normal saline or one of the experimental drug treatments. Rats were returned to the cages containing food pellets for an additional 15 min. They were then placed in the operant conditioning chamber and both levers were inserted. Rats had the opportunity to respond on either lever for 5 min. No outcomes were presented in this session. The following day, rats underwent selective satiety devaluation of the opposite outcome from the previous day, followed by injection of the same drug as was used the previous day, and a choice test. Overall, the devaluation test was repeated six times. Under each drug treatment condition, rats underwent chocolate and grain pellet devaluation to control for pellet preference. Rats received reminder instrumental training sessions between consecutive devaluation sessions.

#### Locomotor Activity Assay

Rats were individually placed in an activity-monitoring arena equipped with an automated locomotor activity detection system (Accuscan, Columbus OH, USA). Rats were placed in the arena for a 30-min habituation session. Immediately after habituation, rats were injected with normal saline and returned to the arena for 30 min. This was followed by an injection of one of the experimental drugs, after which the rats were returned for a final 30-min session. Locomotor activity was estimated based on the number of photobeam breaks that occurred as animals moved through the arena.

### Drug Treatment

All drugs were purchased from Sigma Aldrich (St. Louis, MO, USA) and dissolved in 0.8% normal saline. For the outcome devaluation tests we used a dose of 0.0025 mg/kg for SCH23390, 0.001 mg/kg for Quinpirole, 3.0 mg/kg for SKF38393 and 0.1 mg/kg for Raclopride (D2R-antagonist).

### Statistics and Data Analysis

For instrumental conditioning tests, the rate of response was calculated as the number of lever presses per min during each session. Reinforcer type (chocolate or grain pellet) was collapsed across instrumental training sessions, as no effect of reinforcer type was observed on measures of response rate. For the devaluation test, responses were categorized as “devalued” if the rat made a response on the lever associated with the sated outcome, and “valued” if the rat made a response on the lever associated with the non-sated outcome. Data were normalized by dividing responses on the valued or devalued lever by total (valued plus devalued) responses. Normalization was carried out because of strain differences in overall response rates during the tests. Drug and saline injections were intermixed for all experiments; therefore, there was no injection-order effect to influence outcome devaluation responding. Similarly, chocolate and grain devaluation was counterbalanced across devaluation sessions to control for devaluation flavor-order effects.

Data analysis was conducted using SPSS v20. The normality of data distribution was checked using Kolmogorov–Smirnov tests. All data were normally distributed (*p* > 0.1). To analyze instrumental performance, we used a 2-factor analyses of variance (ANOVA) and planned comparisons using two-tailed *t*-tests. The level of significance was set at *α* = 0.05 throughout our analyses.

To analyze outcome devaluation data, we used mixed-model ANOVAs and planned comparisons using two-tailed *t*-tests. For rats that received SCH 23390 and Quinpirole injections, eight SHR and nine WKY rats received only one devaluation session under each drug condition. The results were compared to animals that received two devaluation sessions (10 SHR and 10 WKY), and the same pattern of results were observed; therefore, we combined the two datasets.

## Results

### Instrumental Training Data

**Figure 3:**
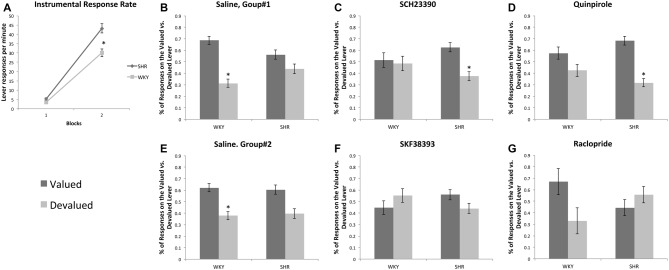
Effects of dopamine receptor agonists and antagonists on extinction test responding following outcome devaluation in SHR and WKY rats. **(A)** SHR displayed a significantly greater response rate during training. Block-1 represents the average of the first three training sessions, and Block-2 represents the average of the last three training sessions showed as the mean number of presses per min on each block of training in SHR (*N* = 35) and WKY (*N* = 36) rats. **(B–G)** Dopamine receptor subtype agonists and antagonists modulate goal-directed behavior in a strain-dependent manner. The percentage of responses on the valued and devalued levers in SHR and WKY under **(B)** Saline; **(C)** SCH23390; **(D)** Quinpirole; **(E)** Saline; **(F)** SKF38393; **(G)** Raclopride (*n* = 5–9 per group; error bars = ±SEM; *significant at *p* < 0.05, paired *t-*tests).

All rats acquired an instrumental response; however, SHR rats exhibited greater response rates across training sessions compared to WKY rats. Figure 3A represents the lever-pressing rate in SHR and WKY rats. A mixed-model ANOVA confirmed: (1) a significant effect of training block (*F*_(1,69)_ = 788.85, *p* < 0.001); (2) a significant effect of strain (*F*_(1,69)_ = 30.137, *p* < 0.001); and (3) a significant block * strain interaction (*F*_(1,69)_ = 25.87, *p* < 0.001). Independent-samples *t*-test showed that SHR’s response rate was significantly higher than WKY’s response rate over the first (*p* = 0.003) and the last (*p* < 0.001) training blocks.

### Outcome Devaluation Results

#### Effects of SCH23390 and Quinpirole on Choice Following Outcome Devaluation (Figures 3B–D)

We injected rats prior to the choice tests with either saline, Quinpirole (0.01 mg/kg) or SCH23390 (0.0025 mg/kg). Responses on the valued and devalued levers were normalized as a percentage of total responses during the test. We carried out separate 3-factor ANOVAs on responses following SCH23390 and Quinpirole treatment using outcome value as the within-subjects factor and strain and type of injection (saline vs. drug) as between-subject factors. These analyses showed significant effects of outcome value for both drugs (SCH23390: *F*_(1,45)_ = 16.63, *p* < 0.001, Quinpirole: *F*_(1,48)_ = 31.67, *p* < 0.001). We also found a significant outcome value * strain * injection interaction for both SCH23390 (*F*_(1,45)_ = 6.1, *p* = 0.017) and Quinpirole (*F*_(1,48)_ = 6.63, *p* = 0.013). These data indicate that the drug effects on responding during devaluation differed depending on the rat strain.

Follow up *t*-tests revealed that following saline injections, WKY rats showed goal-directed behavior by responding at a significantly higher rate on the valued vs. the devalued lever. In contrast, SCH23390 and Quinpirole disrupted goal-directed behavior in these rats as their responses were not significantly different between valued and devalued levers following drug injections. We observed a different pattern in SHR rats. Following saline injections, SHR rats showed an impairment in goal-directed behavior, responding equally on the valued and devalued levers. Both SCH23390 and Quinpirole restored goal-directed behavior in these rats, as shown by significantly greater responding on the valued lever compared to the devalued lever (paired samples *t-*tests, see Figure 3 for significance levels).

#### Effects of SKF38393 and Raclopride on Choice Following Outcome Devaluation (Figures 3E–G)

We injected rats prior to the choice tests with either saline, SKF38393 (3.0 mg/kg) or Raclopride (0.1 mg/kg). We carried out separate 3-factor ANOVAs for SKF38393 and Raclopride using outcome value as the within-subjects factor and strain and type of injection (saline vs. drug) as between-subject factors. These analyses showed significant effects of outcome value for both drugs: SKF38393: *F*_(1,30)_ = 4.28, *p* = 0.047, *β* = 0.52, *η*^2^ = 0.13, Raclopride: *F*_(1,28)_ = 5.45, *p* = 0.027, *β* = 0.62, *η*^2^ = 0.16; however, we observed no significant interactions. Follow up *t*-tests revealed that following saline injections, WKY rats showed goal-directed behavior by responding at a significantly higher rate on the valued vs. the devalued lever. In contrast, following SKF38393 and Raclopride injections we observed responses that were not significantly different between valued and devalued levers in WKY rats. SHR rats showed no significant difference on valued vs. devalued responding following saline, SKF38393 or Raclopride. These data suggest, unlike the effects of Quinpirole and SCH23390, SKF38393 and Raclopride do not improve goal-directed behavior in SHR rats.

The effects of the various drugs on responding during the test following devaluation are not due to effects on food consumption or response rates more generally. We found no effect of drug type on the amount of food consumed during the satiety test (see “Food Consumption” section). Likewise, the effects on goal-directed behavior cannot be explained by an inability to produce a motor response, as all animals included in the test responded on the lever (see “Locomotor Activity Test” section).

#### Goal-Directed Score

**Figure 4:**
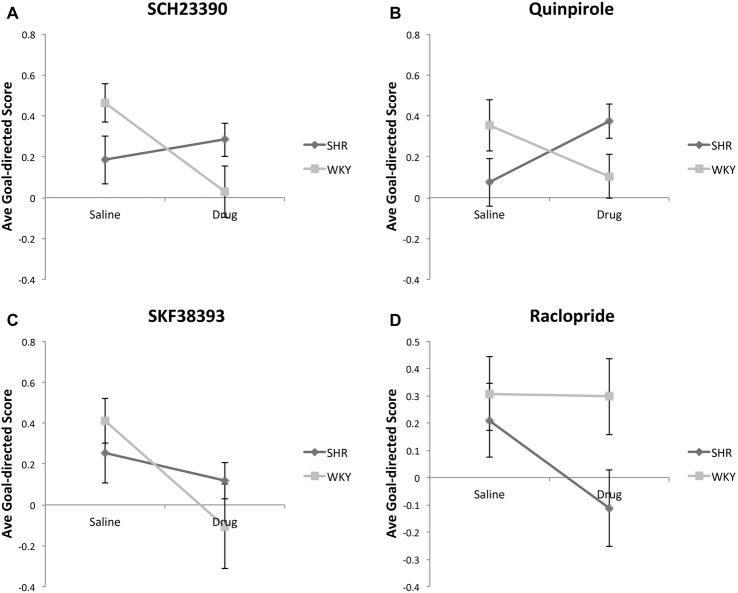
Effects of dopamine receptor agonists and antagonists on Goal-directed Score (GDS) following outcome devaluation in SHR and WKY rats. GDS was calculated using the formula: [% of valued responses − % of devalued responses)/(% of valued responses + % of devalued responses)]. Dopamine receptor subtype agonists and antagonists modulate GDS in a strain-dependent manner. The GDS under injections with normal saline or: **(A)** SCH23390; **(B)** Quinpirole; **(C)** SKF38393; **(D)** Raclopride; (*n* = 4–9 per group; error bars = ±SEM; significant drug × strain interactions were observed under SCH23390 (1) and Quinpirole **(B)**, paired *t*-tests).

For this analysis, we calculated a Goal-directed Score (GDS) for each animal using the following formula: [(% of valued responses −% of devalued responses)/(% of valued responses +% of devalued responses)]. Figure 4 shows the average GDS in SHR and WKY rats under Figure 4A SCH23390, Figure 4B Quinpirole, Figure 4C SKF38393 and Figure 4D Raclopride, as compared to normal saline. Mixed-model ANOVAs were conducted for each drug using type of injection (drug vs. normal saline) as within-subject factor and strain as between-subject factor. These analyses revealed significant drug * strain interaction for SCH23390 (*F*_(1,12)_ = 4.93, *p* = 0.046) and Quinpirole (*F*_(1,14)_ = 8.04, *p* = 0.013) and significant drug effect for SKF38393 (*F*_(1,10)_ = 7.7, *p* = 0.02). Overall, these data indicate that the effects of SCH23390 and Quinpirole on goal-directed responding differ by strain: in SHR rats these drugs tend to increase goal-directed responding, whereas in WKY rats they tend to decrease goal-directed responding.

#### Total Response Rate Under Different Drug Status During the Devaluation Test

##### Effects of SCH23390 and Quinpirole on Response Rate Following Outcome Devaluation

**Figure 5:**
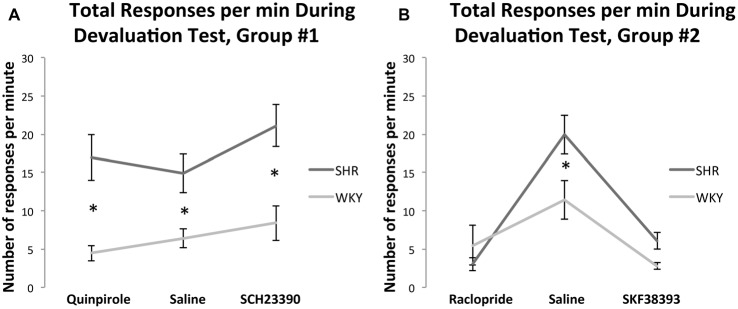
Total responses per minute during devaluation test under injections with **(A)** normal saline, Quinpirole, or SCH23390 in SHR (Quinpirole *N* = 8, saline *N* = 16, SCH *N* = 9) and WKY (Quinpirole *N* = 10, Saline *N* = 18, SCH *N* = 6) rats and **(B)**: normal saline, Raclopride, or SKF38393 in SHR (Raclopride *N* = 9, saline *N* = 9, SKF *N* = 8) and WKY (Raclopride *N* = 5, Saline *N* = 9, SKF *N* = 5) rats (error bars = ±SEM; *significant at *p* < 0.05).

Figure 5A shows the effect of Quinpirole and SCH23390 compared to normal saline injections on total responses per minute (the average of responses on both levers per minute) for both rat strains during the devaluation test. A 2 × 3 multifactorial ANOVA with total responses per minute as dependent variable and strain and medication status as fixed factors showed no significant effects of drug (*F*_(2,61)_ = 1.76, *p* = 0.18) or strain * drug interaction (*F*_(2,61)_ = 0.65, *p* = 0.52). However, there was a significant effect of strain (*F*_(1,61)_ = 34, *p* < 0.001). Overall, these results indicate that drug injections did not affect total response rate during the devaluation test.

##### Effects of SKF38393 and Raclopride on Response Rate Following Outcome Devaluation

Figure 5B shows the effect of SKF38393 and Raclopride compared to normal saline injections on the total responses per minute during the devaluation test. A 2 × 3 multifactorial ANOVA with total responses per minute as dependent variable and strain and medication status as fixed factors showed significant effects of drug (*F*_(2,42)_ = 25.65, *p* < 0.001) and strain* drug interaction (*F*_(2,42)_ = 4.08, *p* = 0.024) with an effect of strain approaching significance (*F*_(1,42)_ = 4.04, *p* = 0.051). SHR rats showed a significantly greater response rate compared to WKY rats under normal saline (*t*_(16)_ = 2.4, *p* = 0.029), and SKF38393 (*t*_(14)_ = 2.85, *p* = 0.013). Compared to saline, SKF38393 and Raclopride significantly reduced response rates in SHR rats (SKF38393: *t*_(10.93)_ = 5.07, *p* < 0.001; Raclopride: *t*_(9.82)_ = 6.41, *p* < 0.001). In WKY rats, SKF38393 significantly reduced response rates compared to saline (*t*_(8.4)_ = 3.41, *p* = 0.009). Overall, these results indicate that drug injections affected total response rate during the devaluation test in the two strains as compared to responses under normal saline injections. However, in this experiment the first two devaluation sessions were carried out under normal saline, while drug injections were carried out during the last four devaluation sessions. Therefore, since the decrease in response rate was observed under injections of both drugs (SKF38393 [D1-*agonist*] and Raclopride [D2-*antagonist*]), lower sensitivity to the devaluation test, which can ensue with repetitive devaluation sessions, could account for these results. In line with this, locomotor activity test results showed that both drug injections did not affect locomotion at doses of 3.0 mg/kg SKF38393 or 0.1 mg/kg Raclopride (see “Locomotor Activity Test Results” section).

#### Exclusion Criteria

Exclusion criteria for analysis included: (1) Outcome preference: we excluded rats that had a preference to chocolate or grain pellets during the last block of instrumental training with a response rate of >1.5× responses per minute on the lever that is associated with the preferred outcome. Out of 72 rats, 8 SHR rats and 5 WKY rats were excluded from the outcome devaluation test. (2) Lever preference: we excluded rats that had a preference to the right or left lever during the extinction test with >2 SD from the mean percentage valued or devalued. We excluded two devaluation sessions of SHR and four devaluation sessions of WKY rats. (3) Low response rate: we excluded rats that had a low response rate during extinction with a total response of <1 per minute on both levers. We excluded 13 devaluation sessions of WKY rats and one devaluation session of SHR rats. See Table 1 for reference.

**Table 1:** Number of rats included across strain and medication status.

Drug	Strain	Exclusion	Final N
Normal saline	SHR	8 pellet preference; 2 lever preference; 1 no training	25
	WKY	5 pellet preference; 3 low response rate; 1 no training	27
Quinpirole	SHR	4 pellet preference	8
	WKY	2 pellet preference	10
SCH23390	SHR	3 pellet preference	9
	WKY	3 pellet preference; 2 low response rate; 1 no training	6
Raclopride	SHR	2 pellet preference; 1 no training	9
	WKY	1 pellet preference; 2 lever preference; 4 low response rate	5
SKF38393	SHR	2 pellet preference; 1 low response rate; 1 no training	8
	WKY	1 pellet preference; 2 lever preference; 1 low response rate	8

### Food Consumption

To determine whether drug or rat strain influenced food consumption during the devaluation procedure, we examined the amount of food rats consumed during the first 40 min of the satiety procedure prior to injections as well as in the 20 min after injections. The majority of food consumption (~90%) occurred during the first 40 min prior to injections (Table 2). In this interval we observed no strain differences in the amount of food consumed. Food consumption following drug injection did not differ from saline except for SKF38393 in SHR rats (*t*_(33.3)_ = 2.64, *p* = 0.012). Since the majority of food consumption occurred during the first 40 min prior to injections (92%), only 8% of food consumption (average of 1.1 g) occurred under the effect of drugs, which implicates that even though there was a significant effect of drugs on food consumption, it was minimal, with a low effect size (*η* = 0.047).

**Table 2:** Amount of food pellets consumed during satiety-induced devaluation after 40 min, and in the 20 min following injections with normal saline, SCH23390, Quinpirole, Raclopride and SKF38393.

Consumption	Strain	All injections	Normal saline	Quinpirole	SCH23390	Raclopride	SKF38393
After 40 min	SHR	14 g ± 5.8	13.7 g ± 6	10.1 g ± 2	10.7 g ± 2.6	15.9 g ± 5.6	15.1 g ± 6.5
	WKY	13 g ± 6.4	12.8 g ± 6.4	9.2 g ± 1.6	8.6 g ± 1.6	15.5 g ± 6.5	13 g ± 7.1
During the 20 min	SHR	1.6 g ± 2	1.2 g ± 1.3	2.0 g ± 2.7	2.3 g ± 1	3 g ± 2.5	0.3 g ± 0.6
after injection	WKY	1.2 g ± 1.7	1.3 g ± 1.7	2.7 g ± 2.3	1.4 g ± 2.6	1.3 g ± 1.3	0.3 g ± 0.6

### Locomotor Activity Test

We recorded locomotor activity during 30-min sessions of habituation, following saline injection, and following drug injection. The following doses were used: SCH23390 (0.0025 mg/kg); Quinpirole (0.001 mg/kg or 0.01 mg/kg); SKF38393 (1.0 mg/kg or 3.0 mg/kg); Raclopride (0.05 mg/kg or 0.1 mg/kg). Horizontal activity was averaged across 5-min blocks for each session (blocks 1–6).

#### Effects of SCH23390 and Quinpirole on Locomotor Activity (Figures 6A–D)

SHR rats traveled a greater distance as measured by horizontal activity during habituation and under saline and drug injections. We analyzed locomotion under each drug separately using two-factor ANOVA’s, with strain and phase (habituation, saline, or drug) as factors. Under both SCH23390 and Quinpirole, we observed a significant effect of strain (SCH23390: *F*_(1,10)_ = 82.31, *p* = 0.025; Quinpirole: (*F*_(1,10)_ = 21.72, *p* = 0.001), indicating SHR rats were traveling a greater distance. Under virtually every condition tested SHR rats made significantly greater movement compared to WKY rats (independent-samples *t*-test: habituation (*t*_(22)_ = 3.36, *p* = 0.003); saline (*t*_(15.72)_ = 4.81, *p* < 0.001), SCH23390 0.0025 mg/kg (*t*_(10)_ = 4.31, *p* = 0.002), Quinpirole 0.001 mg/kg (*t*_(10)_ = 2.33, *p* = 0.042); Quinpirole 0.01 mg/kg (*t*_(6.2)_ = 7.92, *p* < 0.001).

**Figure 6:**
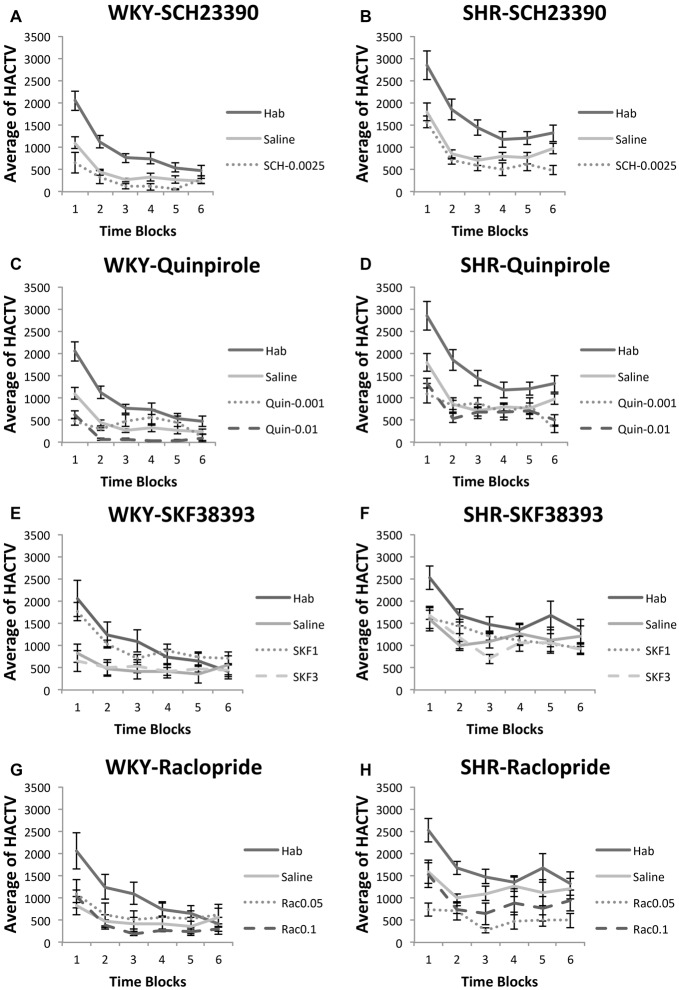
Effects of dopamine receptor agonists and antagonists on locomotor activity. Locomotor activity in WKY (left column) and SHR (right column) is shown in 5-min blocks during habituation, saline injections and drug injections **(A–B)** SCH23390, 0.0025 mg/kg; **(C–D)** Quinpirole, 0.001 mg/kg and 0.01 mg/kg; **(E–F)** SKF38393, 1.0 mg/kg, 3.0 mg/kg **(G–H)** Raclopride, 0.05 mg/kg, 0.1 mg/kg (error bars = ±SEM; *n* = 6–12 per group; paired *t*-tests; HACTV = average horizontal activity).

We observed a significant effect of phase for both drugs (SCH23390 *F*_(2,20)_ = 21.22, *p* < 0.001; Quinpirole (*F*_(3,30)_ = 56.76, *p* < 0.001). Comparisons to saline revealed marginally reduced locomotion in rats exposed to 0.0025 mg/kg SCH23390 in both WKY (paired-samples *t*-test: *t*_(5)_ = 2.24, *p* = 0.076) and SHR (*t*_(5)_ = 2.24, *p* = 0.075). Under 0.001 mg/kg Quinpirole, we observed no overall effect on locomotion in WKY (*t*_(5)_ = 0.17, *p* = 0.83), and a marginally decreased locomotion in SHR rats (*t*_(5)_ = 2.18, *p* = 0.08). However, a dose of 0.01 mg/kg did significantly decrease locomotion in both strains as compared to saline injections (WKY: *t*_(5)_ = 5.99, *p* = 0.002, SHR: *t*_(5)_ = 2.61, *p* = 0.047).

##### Effects of SKF38393 and Raclopride on Locomotor Activity (Figures 6E–H)

We again observed greater distance traveled in SHR rats. Under SKF38393 and Raclopride we observed a significant effect of strain (ANOVA: SKF38393 (*F*_(1,10)_ = 11.33, *p* = 0.007; Raclopride (*F*_(1,10)_ = 5.79, *p* = 0.037). Locomotion was significantly greater in SHR rats as compared to WKY rats during virtually every condition (independent-samples *t*-tests: habituation (*t*_(10)_ = 2.28, *p* = 0.046); saline (*t*_(10)_ = 2.94, *p* = 0.015), SKF38393 3.0 mg/kg (*t*_(10)_ = 3.89, *p* = 0.003); Raclopride 0.1 mg/kg (*t*_(10)_ = 2.3, *p* < 0.045) injections.

We observed a significant effect of phase for both drugs (SKF38393 *F*_(3,30)_ = 10.18, *p* < 0.001; Raclopride *F*_(3,30)_ = 10.84, *p* < 0.001). Within-subjects comparisons revealed no significant differences from saline in animals treated with SKF38393 (all *t*-tests n.s.). Among rats treated with raclopride, no significant differences from saline in locomotor activity were observed except in SHR rats at a dose of 0.05 mg/kg, which significantly decreased locomotion in these animals (*t*_(5)_ = 2.66, *p* = 0.045).

## Discussion

Our results reveal novel roles for dopamine receptor sub-types on goal-directed behavior in rats, and suggest how dysregulation of activity across these sub-types may give rise to behavioral impairments in SHR rats. SHR rats exhibit impaired goal-directed behavior when tested using an outcome devaluation paradigm, replicating our previous findings (Natsheh and Shiflett, 2015). Here, we report that stimulation of D2 receptors or inhibition of D1 receptors restored goal-directed behavior in SHR rats. Conversely, we found that stimulation of D1 receptors or inhibition of D2 receptors did not improve goal-directed behavior in SHR rats. In WKY rats (the normotensive strain most often used as a control group for SHR) we observed goal-directed behavior under control conditions. However, in contrast to SHR rats, treatment with quinpirole and SCH23390 impaired goal-directed behavior. The divergence in response to these drugs on measures of goal-directed behavior between strains suggests fundamental differences in how dopamine signaling is engaged during learning in SHR rats.

Our findings support the notion that balanced activation of D1 and D2 receptors is essential to display goal-directed behavior. Our results are in agreement with models of striatal dopamine function in action selection and initiation (Keeler et al., 2014). Viewed through the lens of dopamine function, some features of ADHD, such as impaired motivation, may be attributed to misbalanced activity between these receptor sub-types. That is, over-activation of D1 receptors at the expense of D2 receptors might account for SHR’s tendency towards reward proximal behavior that is under greater control of the habitual system (Figure 1). Using selective D2 receptor agonists and D1 antagonists restored the balance of activation between dopamine receptor sub-types and hence remediated the deficit in goal-directed behavior in SHR rats, whereas these same drugs caused a misbalance of activation in WKY rats and impaired action control.

We hypothesize that dopamine receptor agonists and antagonists restore goal-directed behavior in SHR rats through their stimulation of “direct” striato-nigral and “indirect” striato-pallidal pathways. Dopamine D1 and D2 receptors are almost exclusively expressed in the direct and indirect pathways, respectively (Albin et al., 1989; Gerfen et al., 1990; Wichmann and DeLong, 1996; Burke et al., 2017). Although these pathways are traditionally associated with motor activity, there is increasing evidence that they are also essential for many aspects of learning (Seger and Cincotta, 2006; Pennartz et al., 2009; Keeler et al., 2014). Phasic and tonic dopamine neuron activity differentially activates the direct and indirect pathway based on the unique binding properties of dopamine receptor subtypes: D1 receptors are stimulated during phasic DA release and exhibit low-affinity binding of DA, while tonic release is responsible for modulating high-affinity D2 receptors. Phasic firing is functionally involved in reward-based behavior (Mirenowicz and Schultz, 1994, 1996; Grace et al., 2007; Hikida et al., 2010; Kravitz et al., 2010, 2012; Yawata et al., 2012; Morita and Hikida, 2015). The indirect pathway has been involved in behavioral flexibility through inhibiting actions in reward learning paradigms, leading to flexibly switching between behaviors (Hikida et al., 2010; Kravitz et al., 2012; Yawata et al., 2012). Reduced inhibition of the indirect pathway, through low stimulation of D2R, can lead to loss of inhibitory control, resulting in behavioral deficits such as compulsivity, impulsivity, or excessive habit formation (Yin et al., 2004; Johnson and Kenny, 2010; Seger and Spiering, 2011; Bock et al., 2013). SHR rats have a high density of striatal D1 dopamine receptors and atypical D2 dopamine receptor activity (Lim et al., 1990a,b; Yu et al., 1990; Linthorst et al., 1993; Russell et al., 1995; Carey et al., 1998). Although it remains to be tested, our findings suggest that misbalanced activation exists between the direct and indirect pathway in SHR rats as a consequence of this differential distribution of D1 and D2 receptors.

The acquisition and deployment of goal-directed behavior has been shown to depend on a balanced activity in direct and indirect pathway neurons that may be weighted toward the indirect pathway and the inhibition of its functional output (Macpherson et al., 2014; Shan et al., 2014). Accordingly, normal corticostriatal function should represent a balanced activation/inactivation in the direct and the indirect pathways. Dopamine depletion in the striatum, such as in Parkinson’s disease, results in hypo-activation/hypo-inhibition of the direct/indirect pathways, respectively that account for motor dysfunction (Galvan and Wichmann, 2008; Magrinelli et al., 2016), as well as cognitive deficits (Frank et al., 2004; Redgrave et al., 2010; de Wit et al., 2011). Some studies have proposed that the motor hyperactivity in ADHD may reflect a “reverse Parkinsonism” that is characterized by either overstimulation of dopaminergic activity in the direct pathway, or excessive dopaminergic inhibition of the indirect pathway (Castellanos, 1997). Here, we argue that specific dopamine receptor modulation of the direct and indirect pathways might explain motivational impairments in ADHD, in addition to motor symptoms. Evidence suggests that naturally occurring polymorphisms of the D1R and D2R genes are implicated in patients with ADHD; however, data on the functional significance of specific polymorphisms of these two genes are still inconclusive (Rowe et al., 1999; Bobb et al., 2005; Serý et al., 2006; Luca et al., 2007; Ribasés et al., 2012).

The effects of dopamine modulation on rats’ performance in the devaluation paradigm are not likely a consequence of memory modulation. It has been previously shown that instrumental incentive learning is not dopamine dependent (Dickinson et al., 2000; Wassum et al., 2011). Thus, animals should have intact memory of the incentive properties of the instrumental outcomes during the choice test. It could be argued that the lack of sensitivity to outcome value in SHR rats may reflect either a performance deficit or a learning/memory deficit. However, SHR rats that received D2R agonist or D1R antagonist injections following outcome devaluation prior to the choice test show value-sensitive responding. Therefore, these animals did encode action-outcome associations during instrumental learning; however, they were only able to use these associations to guide behavior when tested under the effects of D2R stimulation or D1R inhibition. Thus, the deficits we observed in tests of goal-directed behavior in non-medicated SHR rats likely reflect a deficit in performance and not learning of goal-directed actions. Similarly, we previously found that methylphenidate was effective in remediating goal-directed behavior in SHR (Natsheh and Shiflett, 2015). Our current results suggest that increased activation of D2R in response to methylphenidate may have been responsible for this effect. Methylphenidate is known to increase dopamine availability in the striatum (Volkow et al., 2002; Wilens, 2008). Given D2R’s higher affinity to dopamine (Marcellino et al., 2012), we expect that methylphenidate exerts its behavioral effects by increasing D2R activation (inhibiting the indirect pathway), rather than D1R activation (activating the direct pathway). D2 receptor expression in the striatum of ADHD patients is correlated with trait motivation, further supporting the relationship between D2 activity and motivational impairments in ADHD (Volkow et al., 2011).

Although the striatum is clearly involved in action control, many studies have shown that other brain regions might also be implicated in this behavior. Lesions in the prefrontal cortex significantly decreased sensitivity to outcome devaluation in healthy animals (Hitchcott et al., 2007). Dopamine D1 and D2 signaling was shown to influence prefrontal circuits that guide goal-directed behavior. Specifically, a balanced activation of prefrontal D1R and D2R seems to be essential for optimal temporal expectations and cognitive flexibility during action control processes (St. Onge et al., 2011; Parker et al., 2013, 2015). Further, altering neural excitation of the thalamostriatal pathway produced a deficit in goal-directed behavior (Bradfield et al., 2013; Okada et al., 2014; Aoki et al., 2015). Another study revealed that altering the connection between the basolateral amygdala and the nucleus accumbens impairs sensitivity to outcome value during instrumental responding (Shiflett and Balleine, 2010). Thus, future studies are essential to characterize region-specific influence of D1R and D2R activation in SHR rats during instrumental performance.

Although SHR is the most commonly used and most widely accepted rat model of ADHD (Sagvolden et al., 1992, 1993), utilizing an animal model to study neurobehavioral aspects of diseases has many limitations. It is impossible for an animal model to completely portray all characteristics of a disorder. Further, given that the pathophysiological mechanisms of ADHD are still unclear, it is especially difficult to find an animal model that will fully match its neurobehavioral correlates. Another limitation of our study was examining medication modulation in SHR and WKY rats under acute administration (one dose). Many studies have reported significant variations in the therapeutic effects of acute vs. chronic treatment with dopaminergic medications. For example, the effects of quinpirole at low doses may primarily activate pre-synaptic D2 autoreceptors, causing an increase in DA release, whereas at higher doses quinpirole may primarily affect post-synaptic D2 receptors. Future studies with different treatment time points are required to address this issue.

In conclusion, we show for the first time that the dominant habitual response in SHR rats might be due to an over-activation of D1R (over-activation of the direct pathway) and/or under-activation of D2R (hypo-inhibition of the indirect pathway). Modulating dopamine receptor activity has a clear effect on mediating action control behavior in SHR and WKY rats. Thus, unraveling action control mechanisms in ADHD can broaden our understanding of the neural circuits underlying cognitive symptoms of this disorder. Further studies using pharmacological and neural imaging techniques in patients with ADHD are imperative to delineate behavioral and neural action control mechanisms as well as novel treatment options for this disorder.

## Author Contributions

JN and MS developed the research hypothesis tested in this study. JN carried out the experiments and wrote the manuscript with supervision, feedback and critical revisions from MS.

## Conflict of Interest Statement

The authors declare that the research was conducted in the absence of any commercial or financial relationships that could be construed as a potential conflict of interest.
